# Missing the human connection: A rapid appraisal of healthcare workers’ perceptions and experiences of providing palliative care during the COVID-19 pandemic

**DOI:** 10.1177/02692163211004228

**Published:** 2021-03-29

**Authors:** Lucy Mitchinson, Anna Dowrick, Caroline Buck, Katarina Hoernke, Sam Martin, Samantha Vanderslott, Hannah Robinson, Felicia Rankl, Louisa Manby, Sasha Lewis-Jackson, Cecilia Vindrola-Padros

**Affiliations:** 1 Marie Curie Palliative Care Research Department, University College London, London, UK; 2 Institute of Population Health Science, Queen Mary University of London, London, UK; 3 Institute of Epidemiology and Healthcare, University College London, London, UK; 4 Institute for Global Health, University College London, London, UK; 5 Oxford Vaccine Group, Churchill Hospital, University of Oxford, Oxford, UK; 6 School for Policy Studies, Faculty of Social Sciences and Law, University of Bristol, Bristol, UK; 7 Nuffield College, University of Oxford, Oxford, UK; 8 Department of Targeted Intervention and Rapid Research Evaluation and Appraisal Lab (RREAL), University College London, London, UK

**Keywords:** COVID-19, health personnel, palliative care, qualitative research, delivery of healthcare

## Abstract

**Background:**

During infectious epidemics, healthcare workers are required to deliver traditional care while facing new pressures. Time and resource restrictions, a focus on saving lives and new safety measures can lead to traditional aspects of care delivery being neglected.

**Aim:**

Identify barriers to delivering end-of-life care, describe attempts to deliver care during the COVID-19 pandemic, and understand the impact this had on staff.

**Design:**

A rapid appraisal was conducted incorporating a rapid review of policies from the United Kingdom, semi-structured telephone interviews with healthcare workers, and a review of mass print media news stories and social media posts describing healthcare worker’s experiences of delivering care during the pandemic. Data were coded and analysed using framework analysis.

**Setting/Participants:**

From a larger ongoing study, 22 interviews which mentioned death or caring for patients at end-of-life, eight government and National Health Service policies affecting end-of-life care delivery, eight international news media stories and 3440 publicly available social media posts were identified. The social media analysis centred around 274 original tweets with the highest reach, engagement and relevance. Incorporating multiple workstreams provided a broad perspective of end-of-life care during the COVID-19 pandemic in the United Kingdom.

**Results:**

Three themes were developed: (1) restrictions to traditional care, (2) striving for new forms of care and (3) establishing identity and resilience.

**Conclusions:**

The COVID-19 pandemic prohibited the delivery of traditional care as practical barriers restricted human connections. Staff prioritised communication and comfort orientated tasks to re-establish compassion at end-of-life and displayed resilience by adjusting their goals.

**What is already known about the topic?**

- In emergency medical situations the delivery of traditional care can be disrupted by work pressures and infection prevention measures.
- With the death toll rising worldwide, we do not know what impact the COVID-19 outbreak is having on the delivery of end-of-life care.

**What this paper adds?**

- We added to the knowledge base of end-of-life care delivery during infectious epidemics by highlighting how increased work pressure, changing policy, reduced patient interaction and use of PPE make delivering end-of-life care challenging for healthcare workers.
- We have shown that healthcare workers struggled to connect with patients due to increased work pressures and limited opportunities for human interaction.
- We now understand how changes to end-of-life care impact healthcare workers and how they respond by attempting to reintroduce human connection and compassion at the end-of-life.

**Implications for practice, theory or policy**

- Designated teams and processes for communication should be established early as addressing patient loneliness and isolation was a priority for staff.
- Policies on care delivery in epidemic circumstances should support healthcare workers forming human connections with patients and retain the fundamentals of palliative care.

## Introduction

A main tenet of palliative care is providing a ‘good death’ for patients. This is enabled by appropriate symptom management, discussions with patients about their wishes, and the provision of emotional and spiritual support.^1^ This holistic approach to care requires healthcare workers to interact and connect with their patients, however when staff and resources are stretched by epidemics, building connection with patients can be neglected.^2^

During the SARS-CoV-1 outbreak in Singapore, infection prevention measures such as personal protective equipment and patient isolation created feelings of disconnect between healthcare workers, patients and their families.^3^ Reduced staff and resources led to similar consequences during the Ebola outbreak in West Africa.^4,5^ Traditional care delivery can also be impacted when a focus on saving the greatest number overshadows the management of those at the end-of-life.^6^

Healthcare workers can be empowered by delivering care which enhance patient wellbeing while also providing clinical treatment,^7^ but they risk moral distress, unresolved grief and burnout when compassion and human connections are absent.^8^

As COVID-19 spread, healthcare systems worldwide were forced to adapt.^9^ Patient visiting restrictions were implemented to avoid further infection,^10–13^ however these policies often led to distraught family members and instances of staff comforting patients in their final moments.^14–16^ Attempts to mitigate these effects were made through the introduction of video communication to enable goodbyes, however reports suggest these new approaches cannot always be achieved and may be too distressing for families.^17,18^

The need for empathetic human interaction between healthcare workers and patients remains essential during pandemics.^19^ As the number of deaths continues to rise, we need to explore healthcare workers’ experiences to understand how they respond to challenges and work to maintain quality end-of-life care during the COVID-19 pandemic.

## Methods

### Research questions

(1) How do healthcare workers’ perceive end-of-life care during the COVID-19 pandemic? and what barriers do healthcare workers face in delivering end-of-life care?
(2) In which ways do healthcare workers attempt to deliver quality care and provide a good death in emergency circumstances?
(3) How do changes in the provision of traditional end-of-life care affect staff?

*Design*: Data were collected as part of a wider rapid appraisal of healthcare delivery during the COVID-19 pandemic.^20^ A rapid appraisal is a qualitative approach which uses iterative data collection and analysis, to triangulate multiple data sources to understand a situation.^21^ Telephone interviews, a UK policy review, and an analysis of social and mass media (outlined in Table 1), were conducted simultaneously and the data collectively analysed to understand the perspectives and experiences of healthcare workers in the UK. These multiple forms of data collection allow us to consider; how policy influences healthcare workers’ experience of care delivery, how policy changes and additional pressures affect healthcare workers’ thoughts/emotions and whether our findings are reflective of the wider national and international perspective.

**Table 1.** Rapid qualitative appraisal design.^20^

Data source	Method of data collection	Sample	Method of data analysis
Policy review	Policies were selected from legislation.gov.uk, gov.uk, NHS England, NHS Scotland and Public Health England databases and health bodies.	From a total sample of 62, policies published between 1 December 2019 and 1 July 2020, 8 would impact the delivery of end-of-life care so were included in analysis.	Data were extracted into excel and cross-checked by a second researcher who created a conceptual framework to categorise the policies.
Media analysis	Mass media: review of newspaper articles obtained from LexisNexis and a hand search.	Eight international newspaper articles, published between 1 November 2019 and 8 June 2020, contained content relating to healthcare workers experiences of end-of-life care and death during COVID-19.	Data extracted onto a Research Electronic Data Capture (REDCap) form and analysed using framework analysis
	Social media: data were selected using the software Meltwater and sorted into pre-established categories.	3440 social media posts related to end-of-life care and death were gathered from Twitter between 1 December 2019 and 31 May 2020. The conversation centered around 274 original tweets.	Researchers coded selected posts independently.
Healthcare worker interviews	In-depth, semi-structured telephone interviews with a purposive sample of staff from four UK hospitals.	From the wider study sample of 100 interviews, 22 contained content related to end-of-life care or death and were included in analysis. Six additional interviews were conducted with palliative specialists from the same sites to provide specialist knowledge.	Rapid assessment procedure sheets were used to synthesise findings on an ongoing basis and aid familiarisation. Selected transcripts analysed using framework analysis.

*Setting*: The study began during wave 1 of the pandemic in the UK, where access to NHS sites and staff was limited. Due to the high level of activity in large teaching hospitals, three were targeted for initial recruitment. Recruitment also included healthcare workers from other settings who were redeployed to large teaching hospitals during the first wave of the pandemic. This data are reported in separate analyses, as we analysed the early data with the aim of reporting findings in a timely manner.

*Population*: This analysis focuses on data relating to care at end of life, which includes caring for patients who are dying and their families, and work related to planning/preparing for death. For the purpose of this analysis, a healthcare worker was defined as any clinical member of staff who delivered this form of care during the COVID-19 pandemic.

*Sampling*: Staff were purposively sampled to achieve broad coverage of professional roles and levels of experience (sampling strategy outlined in Supplemental Appendix S1). The sample included staff who were working within their usual role and those who had been redeployed. At the time of analysis, 100 interviews had been conducted with healthcare workers from a range of specialities. Members of the research team indicated which interviews addressed the topic of caring for patients at end of life or managing practical and emotional issues relating death, as this was not experienced by all participants. These transcripts were read by LM to determine whether the content related to end-of-life care and would therefore be relevant for inclusion. This process was cross-checked by members of the research team. All interviews identified as containing content relating to end-of-life care at the time of analysis were included. This formed a sample of 22 interviews. An additional six interviews were conducted with palliative care professionals to gain specialist knowledge and to compare the experiences of different professional roles.

*Recruitment*: Clinical leads approached staff to inform them of the study and gather verbal consent to be contacted by the research team. Researchers contacted participants through email and provided the participant information sheet. Informed written consent was given by participants via email and this was confirmed verbally before the interview. Participants had the ability to withdraw consent at any time. The researcher then arranged a telephone interview at a convenient time via email.

### Data collection

#### Telephone interviews

In-depth telephone interviews were carried out with healthcare workers from four UK hospitals between 19 March 2020 and 1 July 2020. Interviews were conducted by researchers from the Rapid Research and Appraisal Lab. Interviewers included PhD and Masters students, post-doctoral researchers, university lecturers and senior research fellows. The semi-structured topic guide (Supplemental Appendix S1) focused on staff experiences and their perceptions of COVID-19 and healthcare delivery. Questions addressing palliative and end-of-life care were added part-way through the interview process to explore this topic in greater depth. By conducting analysis concurrently to data collection, it became apparent that experiences of delivering care at end of life were of great importance to staff as it was frequently discussed unprompted in interviews. Additionally, with the growing number of deaths, the research team acknowledged the benefit of exploring staff experiences and forms of care delivery relating to end of life at this time. Interviews were audio recorded and transcribed verbatim, with key comments documented in notes.

#### UK policy review

A rapid review of UK policies relating to healthcare delivery during the COVID-19 pandemic was conducted. Following a framework for rapid evidence synthesis,^22^ policies were gathered using a search strategy (Supplemental Appendix S2) on multiple databases and websites of health bodies. Policies were screened and included if they were (1) published between 1 December 2019 and 1 July 2020, (2) aimed at healthcare delivery, (3) related to COVID-19 and (4) impacted end-of-life care (e.g. specifically mentions care at end of life or palliative care, or a general policy which would impact all forms of care delivery such as PPE). The identified policies were cross checked within the research team.

#### Mass and social media analysis

A LexisNexis database search (see Supplemental Appendix S2) and a hand search of relevant newspapers and magazines were conducted for articles published between 1 November 2019 and 8 June 2020. Article titles, then full texts were screened. Articles relating to healthcare workers experiences of delivering care at end of life were selected and key information extracted.

Social media data were gathered using keyword searches (see Supplemental Appendix S2) on Twitter, Reddit, Facebook (public groups), Instagram (public accounts) and YouTube from 1 December 2019 to 31 May 2020. Posts were coded into pre-defined categories and duplicates removed.

**Figure 1.**
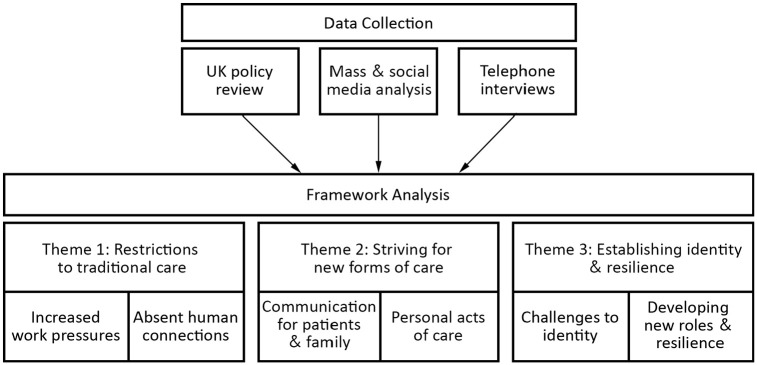
Analysis process with theme and sub-theme structure.

*Data Analysis*: A realist ontological and a critical-realist epistemological position was taken as healthcare workers described their reality, yet these experiences are shaped by their position within it.^23^ Framework analysis was conducted following the methodology of Gale et al.^24^ Rapid Assessment Procedure sheets^20^ were used for familiarisation, then a secondary analysis was conducted on the interviews transcripts which mentioned care at end of life. A sample of five interviews were coded, and the codes grouped to form a coding frame which was applied to the remaining transcripts. After coding, the data was entered into a framework analysis matrix. Each data source (interview, news article, or policy) was entered into a separate row and referred to as a ‘case’.^24^ When reading down the columns, all cases within the same code could be compared to identify patterns. New columns were added to the matrix when new ideas were developed. Triangulation occurred as the separate data sources were compared and the similarities identified. Codes and the corresponding data were grouped together to produced overarching themes. Themes were discussed by the research team (LM, AD, CB and CV) and altered until it was agreed they reflected the core components of the dataset (Figure 1). Member checking processes were implemented where emerging findings were shared with study participants. Semantic discourse and topic analysis were used to understand the most frequently used and weighted keywords or viral hashtags, and to prioritise themes of discussion and clusters of topics, focusing on the UK.

*Ethical Issues*: Ethical approval was granted by the Health Research Authority in the UK (IRAS: 282069; Date: 2 April 2020) and by the hospitals where the study took place. All researchers received NHS Digital and Health Education England training on data security awareness. Interviews were conducted via telephone and were recorded with participants’ consent. Recordings were transcribed verbatim with personal identifiers removed and stored on a secure server. Data analysis included generic categories (e.g. general role instead of job title) which avoided participants being identified through interview quotes.

## Results

Twenty-eight interviews with healthcare workers were included in analysis (Table 2). Four UK policies related directly to end-of-life care were identified,^25–28^ and an additional four policies were included which impacted care delivery.^29–32^ The media search identified eight international news articles^33–40^ and 3440 English language tweets, with the social media conversation focused on 274 original tweets. Three overarching themes were developed: (1) restriction to traditional care, (2) striving for new forms of care and (3) establishing identity and resilience. Additional data extracts are presented in Supplemental Appendix S3.

**Table 2.** Interview participant characteristics.

Age: mean (SD)	39.23 years (7.67)
	Unknown = 2
Gender: female *n* (%)	22 (78.57)
Time qualified: mean (SD)	13.90 years (8.94)
Time qualified: range	1–37 years
	Unknown = 2
Sector (%)	
Secondary and tertiary care	27 (96.43)
Community services	1 (3.57)
Speciality	
Anaesthesia*	8
Palliative care**	6
Intensive care	4
Infectious diseases and respiratory	3
Accident and emergency	2
Oncology	2
Admissions	1
Paediatrics	1
Elderly medicine	1
Professional role	
Dietician	1
Pharmacist	1
Nurse	14
Registrar	2
Consultant	8
Trainee	1
Unknown	1
Redeployed: *n* (%)	11 (39.29)

* Across different specialities; four in theatre/surgery, two in acute medicine, two in intensive care.

** Palliative specialists included a hospital consultant, a community consultant, senior registrar, clinical nurse specialist, nurse team lead and a member of the educating team.

### Theme 1: Restrictions to traditional care

Healthcare workers held strong beliefs about what constitutes quality end-of-life care and a good death. The introduction of policies which restricted visitors until imminent death,^27,31^ placed patients in single rooms^25,29^ or called for staff and family to wear PPE,^25–27,29–31^ meant that traditional care ideals could not be met. Staff perceptions of end of life became widely negative; ‘This is horrible, it’s away from the family, the family cannot hug them, cannot be with them’ (Anaesthetist, Intensive Care Unit (ICU)). Dying alone was an unacceptable outcome reflected in policy, social and mass media posts.^25,33–35^ The principles of dignified and holistic care were emphasised in policy^25,27,28,32^ yet changes in care delivery led some staff to feel they were ‘not providing the best possible care’ (Consultant, Paediatrics) to patients.^39^

#### Increased work pressures

Dramatic increases in workload were caused by rising patient numbers, reduced staff and additional work managing new settings or redeployed staff. These pressures stopped the delivery of ideal care because ‘it physically wasn’t possible to talk to the relatives and provide them reassurance and comfort, and yet that’s all you really want to do, but it was either a choice of talking to them or answering the alarm’ (Nurse, Critical Care). Mirrored in both interviews and mass media reports,^34^ the increased workload led staff to prioritise clinical care tasks over family support and make difficult decisions between monitoring patients or comforting those at end of life.

#### Absent human connections

A palliative clinician described how by building a relationship they would ‘begin understanding what their needs are, what their wishes are and you can put a plan in place to meet them’ (Consultant, Palliative Care). During the pandemic however interaction was limited as patients deteriorated rapidly and were often sedated, which made forming connections difficult. Healthcare workers perceived care as ‘sort of functional’ (Consultant, Diagnostics and Treatment Unit) due to the lack of interaction. Aspects of care, like emotional support, then became ‘a struggle . . .because somehow you feel distanced’ (Sister, Accident and Emergency (A and E)). Staff felt ‘the normal closeness and kindness is diluted’ (Sister, A and E) by personal protective equipment as small talk, smiles and physical touch were blocked. Their ability to connect with patients was also impacted by visiting restrictions, as staff previously learnt about their patients by witnessing family visits and hearing their views.

### Theme 2: Striving for new forms of care

#### Communication for patients and family

COVID-19 was perceived as a ‘lonely disease’ (Anaesthetist, ICU) so healthcare workers prioritised communication. Family updates were initially difficult to arrange but staff ‘found their feet’ (Nurse, Infectious Diseases) when systems were put in place, such as the introduction of family liaison teams. Policies encouraged connecting patients with family through phone and video calls.^25,27^ Mass media stories and Tweets described how technology was used to enable goodbyes.^34,39^ Facilitating these calls became a priority for many healthcare workers as ‘there are still people who can’t have the visitors side of things and that communication is kind of second’ (Consultant, Elderly Medicine). These calls compensated for the visiting restrictions and helped healthcare workers see their patients as individuals again. Frustration arose when calls were not consistently prioritised and patients missed out on saying goodbye: ‘we could have got him on a phone when he was able to talk, but as it was it was too late’ (Consultant, Elderly Medicine). Lack of clarity around who was responsible for facilitating these calls may have contributed to these delays.

#### Personal acts of care

Healthcare workers wanted to address patient distress by offering ‘whatever human comforts you can provide’ (Anaesthetist, Acute Care). They performed personal acts of compassion such as greeting the patient from outside the room so ‘they can just see you briefly’ (Nurse, Palliative Care) before donning the mask, or trying to ‘touch them and say “its ok” even if they are sedated’ (Nurse, ICU). Others described efforts to talk with patients and ‘make them laugh or smile’ (Nurse, Infection Control). To minimise family distress nurses tried to ‘make sure the patient looks nicer’ (Nurse, ICU) before videocalls. Healthcare workers also took on the role of comforting patients at end of life when family could not be present. This was perceived as vitally important with actions taken to ensure they ‘never allowed anyone to die alone. . . we were always in there with them’ (Nurse, Infection Control). Media reports mirrored these stories describing how staff held hands with scared patients in place of their family.^34–37^

### Theme 3: Establishing identity and resilience

#### Challenges to identity

Healthcare workers had a strong sense of professional identity and struggled when care did not align with their perceptions of success and standards of care. Staff in intensive care described how they ‘can feel defeated when everything you’ve done doesn’t work and the patient continues to get sicker’ (Anaesthetist, ICU). Nurses struggled with the changes to care delivery ‘as a gut feeling, as a nurse, you didn’t feel it was right what you were doing’ (Nurse, Palliative Care). Palliative specialists who would do anything to enable a good death and perceived themselves as ‘rule breakers’ (Nurse, Palliative Care) were now required to enforce policies which contradicted their care beliefs. Also reflected in news articles were feelings of helplessness^38,40^ from the ‘unpredictable’ (Nurse, Infectious Diseases) nature of the illness and working outside their ‘comfort zone’ (Sister, A and E). Staff from all specialities were distressed witnessing how traditional end-of-life care was ‘just taken away from the patients as well and the relative’ (Nurse, ICU). In news reports healthcare workers described seeing patients die without their family as ‘killing all of us inside’^33^ and the end of life was labelled as ‘inhumane’ on social media.

#### Developing new roles and resilience

Some healthcare workers found satisfaction in striving to provide compassionate care. A consultant described collaborating with the Hatzalah (a Jewish emergency medical service) to meet a family’s wishes for a patient to die at home. Despite not succeeding the consultant found the work ‘very satisfying’ (Senior Registrar, Palliative Care) as the family was grateful for their effort. Prioritising tasks which were meaningful to patients and their families helped staff accept difficult situations as ‘what ended up being a comfort for me was that actually they [the family] were okay with that’ (Nurse, Palliative Care). Redeployed staff who lacked specialist skills ‘took ownership’ (Nurse, ICU) of care tasks such as comfort, mouth care, patient appearance and video calls.^34,36,37,39^ On social media, healthcare workers shared learning resources to improve end-of-life care delivery nation-wide. Some staff began to see themselves as an ‘extended family member’ (Nurse, Infection Control) and would advocate for patients in place of their family. They were motivated to provide care as ‘if this was my mum, what would I want them to do. . .we are going to look after them like they are our own’ (Nurse, Infection Control). Adjusting their role and striving to deliver new forms of care appeared to empower staff and offset some of the distress they faced.

## Discussion

### Main findings

During the COVID-19 outbreak, policies and infection prevention measures limited healthcare workers’ ability to provide traditional end-of-life care. Reduced opportunity for interaction made it difficult to connect with patients, and staff felt pressure to prioritise clinical tasks. To address this loss of connection, healthcare workers focused on improving communication for patients and family. They also demonstrated personal acts of care by engaging in small talk, offering reassuring touch and improving patient appearance. Most importantly, staff comforted patients at end of life when family could not be present. Ensuring no one died alone was perceived as the duty of healthcare workers and an essential part of a good death. The changes in care appeared to affect healthcare workers’ sense of identity as it contradicted their beliefs around quality care. Despite initial difficulties, staff adjusted their goals to focus on tasks considered most important to patients and their families, potentially developing staff resilience. While the loss of traditional elements of care were distressing for staff, actively engaging in new forms of human connection and care could be empowering.

### Strength and weaknesses

To our knowledge, this is the first qualitative study to explore how healthcare workers have responded to changes in end-of-life care during the COVID-19 pandemic. This work contributes to the literature on care delivery in pandemic circumstances. The prospective study design helped increase the validity of the findings, as compassion at end of life was raised unprompted by healthcare workers in a large sample of interviews and was a highly discussed topic in the mass and social media analysis. The design also allowed for healthcare workers’ experiences to be captured during the first wave of the pandemic in the UK and contribute to future response efforts.

A potential weakness is the transferability of the findings. Our sample contained a greater proportion of nurses which may have influenced the findings. It is likely however that nurses dealt with the greatest proportion of end-of-life care involving direct patient contact, as palliative care doctors took on the advisory role. Including nurse’s experiences was essential. Most of our participants were from large, London-based hospitals as the research team had greater access and support recruiting at these sites. As data collection occurred during wave 1 of the pandemic, access to NHS sites and staff was restricted. Additionally ethnicity data was not routinely collected at the beginning of the study. The experiences and perceptions of healthcare workers of all ethnicities and across the UK may not have been captured in our data. For example, the availability of PPE was not an issue for our participants, however, reports of limited resources were made elsewhere in the UK.^41–44^ To capture a wider range of experiences, we included descriptions from UK and international media and cross-UK social media accounts to verify our findings. Lastly, the use of telephone interviews raises a potential bias, as interviewers may have missed non-verbal cues or felt less able to explore sensitive topics. However, given the circumstances, telephone interviews remained an appropriate method for capturing rich descriptions of healthcare workers’ experiences.

### What this study adds

In previous emergency situations, traditional care has been restricted by infection prevention measures, increased workload, and limited resources.^2–6^ We have shown that during the COVID-19 outbreak in the UK, healthcare workers faced similar difficulties which led to drastic changes in care provision. While these changes were difficult for patients and families, the impact on healthcare workers had not been previously explored.

Healthcare worker’s felt that patient care was impacted by their inability to form relationships with patients. Patients were less able to communicate their needs and healthcare workers struggled to relieve their suffering. This in turn risked destabilising their professional identity and caused distress. Warnings of compassion fatigue and burnout are frequent in pandemic situations, as healthcare workers can adopt the trauma and stress of those they care for.^45,46^ Research suggests that witnessing patient suffering and feeling unable to prevent it can have lasting psychological effects.^47^ Stories of healthcare workers’ frustration and grief at care delivery have been reported worldwide throughout the pandemic.^14^ Our participants indicated that caring for patients at the end of life in place of their families took an emotional toll.

However, our study also suggests that healthcare workers were empowered by engaging in new forms of care. Human and empathetic interactions have been encouraged as a way to alleviate patient distress in pandemics. Davies and Hayes^19^ recommend engaging with patients through general ‘chit-chat’ and using non-verbal methods such as ‘thumbs up’ gestures and touch (when safe). These human elements were personally instigated by healthcare workers to re-establish human connection and improve the quality of care. Healthcare workers indicated that engaging in these individual behaviours and feeling supported by official processes, helped improve feelings of self-efficacy and appeared to enable them to develop resilience.

While UK policies stated that holistic and compassionate end-of-life care was a priority, the guidance did not address how healthcare workers should establish meaningful connections with patients. Policy provided substantial guidance on managing the symptoms of COVID-19 and palliative care staff noted that this was not difficult. However while the practical aspects of care remained manageable for staff, the holistic components were lacking. Recommendations should be made on how to overcome infection prevention barriers, such as safely showing your face prior to donning PPE, engaging in conversation and using physical touch when appropriate. Renzi et al.^48^ emphasised the importance of small compassionate gestures to patients isolated by COVID-19, such as offering reassurance to patients or providing time and listening.

UK guidance encouraged the use of technology to connect patients and their families^49^ yet staff frequently reported difficulties facilitating these calls due to a lack of clarity in responsibility, technical issues and varying priority between individuals and wards. Updating and supporting families over the phone was also a challenge. In future outbreaks, teams responsible for facilitating daily communication between patients and families should be established early. Redeployed staff who lack specialist skills could take ownership of this and may build resilience by developing a clear role and supporting tasks valued by patients. Designated teams such as family liaison officers have been used to provide daily compassionate updates to family, removing the burden from ward staff and ensuring that human care is not neglected.^50^

Future research should explore patient and family experiences of care during the COVID-19 pandemic. Healthcare workers in our study were distressed by the quality of care delivered and worried about the impact it was having on patients. Understanding their experiences will ensure that future guidance can focus on encouraging the most effective and beneficial elements of care to continue. The use of validated scales to assess aspects of self-efficacy and staff distress may be beneficial. Additionally, investigation into the impact on compassion in care may also be warranted.

## Supplemental Material

sj-docx-1-pmj-10.1177_02692163211004228 – Supplemental material for Missing the human connection: A rapid appraisal of healthcare workers’ perceptions and experiences of providing palliative care during the COVID-19 pandemic Click here for additional data file. Supplemental material, sj-docx-1-pmj-10.1177_02692163211004228 for Missing the human connection: A rapid appraisal of healthcare workers’ perceptions and experiences of providing palliative care during the COVID-19 pandemic by Lucy Mitchinson, Anna Dowrick, Caroline Buck, Katarina Hoernke, Sam Martin, Samantha Vanderslott, Hannah Robinson, Felicia Rankl, Louisa Manby, Sasha Lewis-Jackson and Cecilia Vindrola-Padros in Palliative Medicine
