# Supplementary material for: Missing the human connection: A rapid appraisal of healthcare workers’ perceptions and experiences of providing palliative care during the COVID-19 pandemic
Source: Palliat Med. 2021 Mar 29;35(5):852–61. doi: 10.1177/02692163211004228 (PMC8114443; doi:10.1177/02692163211004228)
Supplement: sj-docx-1-pmj-10.1177_02692163211004228 – Supplemental material for Missing the human connection: A rapid appraisal of healthcare workers’ perceptions and experiences of providing palliative care during the COVID-19 pandemic [file sj-docx-1-pmj-10.1177_02692163211004228.docx]

Title: Appendices for the article ‘Missing the human connection: A rapid appraisal of healthcare workers’ perceptions and experiences of providing palliative care during the COVID-19 pandemic’

Description: *Further methodological detail on the sampling strategy for telephone interviews and interview topic guide. The full search strategies for the social media, mass media, and UK policy reviews are included. Additional data extracts from each workstream are presented within the themes.*

Article Title: Missing the human connection: A rapid appraisal of healthcare workers’ perceptions and experiences of providing palliative care during the COVID-19 pandemic

Author Names: Lucy Mitchinson, Anna Dowrick, Caroline Buck, Katarina Hoernke, Sam Martin, Samantha Vanderslott, Hannah Robinson, Felicia Rankl, Louisa Manby, Sasha Lewis-Jackson and Cecilia Vindrola-Padros.

Appendix 1: Further methodology detail for telephone interviews

*Sampling framework:*

The initial sampling framework for interviewed healthcare workers included a total of 60 participants: 10 emergency department consultants, 10 emergency department nursing staff, 5 emergency department management staff, 10 infection control staff, 5 microbiologists, 10 critical care consultants, 10 nurse consultants. 40 additional participants were purposively sampled based on their role and experience. Out of the 100 interviews, 22 were selected for their content relating to end-of-life care. Six additional interviews were conducted with professions from palliative care departments to provide specialist knowledge. The total number of interviews included were 28.

**Table 3.** Summary of interview topic guide

| **Main question** | **Summary of topics covered by probing questions** |
| --- | --- |
| - Respondent information | Gender; age; time in service; education level; role; ethnicity; sector and type of facility; location of facility |
| - Can you tell me about your role? | Daily tasks; department; responsibilities |
| - Have you been in contact with patients who had suspected and/or confirmed COVID-19? | In what capacity staff had been in contact with COVID-19 patients; how they found working with them; emotional and psychological effects; the effects of PPE on delivering care |
| - How has the COVID-19 outbreak affected health services in your department? | Effect on staff daily tasks and ability to deliver care; cancellation of elective surgeries; isolation of suspected and confirmed cases; impact on the supply of drugs and equipment; redeployment of staff |
| - What were the preparedness strategies implemented locally? | Whether they felt these strategies were enough; what was successful; what should have been prepared differently; training; guidance |
| - Do you currently have any concerns or fears? | In relation to the national effort; in relation to their own work (response efforts, PPE, services) |
| - Over the past months, have you experienced any problems with aspects of your daily life? | Sleeping; eating; concentration; additional worries or anxiety |
| - Have you been provided with mental health support? | Are they aware of support available; have they had the opportunity to speak about their mental health; worrying experiences; interactions between colleagues |
| - Have you been involved in caring for patients who are dying or expected to die soon? | Tasks and responsibilities related to advanced care planning, symptom management, comfort, end-of-life decision making, communicating with families; difficulties and challenges; emotional impact on staff; training and support available; communicating with family members; differences to normal palliative care; how much choice patients had; rules and policies |
| - What do you feel is most important to offer COVID-19 patients at end-of-life and their families? | What was working well; what can be improved; what support needed to be offered to staff delivering palliative care; bereavement support to families |
| - How have health services been strengthened, or how could they be strengthened during the outbreak? | Support to staff from health system and partners; capacity for rapid response; policies and emergency protocols; maintaining normal services; general practice health promotion and community engagement; linkage to support organisations |
| - Is there anything you feel should be changed to make health services more effective in future emergencies? | Support to staff from other sources; coordination and official guidance of COVID-19 response; early detection and reporting; volunteers; disease outbreak control activities; testing public and staff |
| - Specific questions related to other sub-analyses | Experiences in relation to gender, race, ethnicity; home life; caring responsibilities, pregnancy |

Appendix 2: Search strategies and screening criteria

*Social media review Boolean search terms:*

((bio:"healthcare professional" OR bio:"healthcare worker" OR bio:"doctor" OR bio:"NHS" OR bio:"nurse" OR bio:"physio*" OR bio:"Paramedic" OR bio:"Ambulance work*" OR bio:"Ambulance driver*" OR bio:"Occupational Therapist") AND ("coronavirus" OR "#coronavirus" OR “corona” OR "COVID-19" OR "COVID 19" OR "COVID19" OR "#COVID19" OR "COVID_19" OR "COVID" OR "severe acute respiratory syndrome coronavirus 2" OR "severe acute respiratory syndrome coronavirus 2" OR "2019-nCoV" OR "SARS-CoV-2" OR "2019nCoV") AND ("palliative*" OR "end of life"))

*Mass media review*

*Search terms:* ("healthcare professionals"[All Fields] OR "healthcare worker"[All Fields] OR "doctor"[All Fields] OR "nurse"[All Fields]) AND (("coronavirus"[MeSH Terms] OR "coronavirus"[All Fields]) OR corona[All Fields] OR ("COVID-19"[All Fields] OR "severe acute respiratory syndrome coronavirus 2"[Supplementary Concept] OR "severe acute respiratory syndrome coronavirus 2"[All Fields] OR "2019-nCoV"[All Fields] OR "SARS-CoV-2"[All Fields] OR "2019nCoV"[All Fields] AND ("coronavirus"[MeSH Terms] OR "coronavirus"[All Fields])) AND 2019/12[PDAT] : 2030[PDAT])))

*Criteria:*

1. Focus on the perspectives or experiences of healthcare workers (self-reported or narrated in third person)
2. Focus on the response strategy aimed at COVID-19
3. Published from November 2019 to 8 June 2020
4. Published in English.

*UK policy review*

*Search terms:* COVID-19 OR coronavirus AND palliative care

*Criteria:*

1. Published from 1 November 2019 to 1 July 2020;
2. Aimed at healthcare delivery (i.e. not focusing on prevention, social isolation, etc);
3. Related to the COVID-19 pandemic;
4. Related to the delivery of palliative care.

Appendix 3: Additional data extracts

**Table 4.** Data extracts per workstream within themes

|  | **Theme 1: Restrictions to traditional compassionate care** | **Theme 2: Striving for new acts of compassion** | **Theme 3: Establishing identity and resilience through compassion.** |
| --- | --- | --- | --- |
| **Mass Media:** | A sister working on the intensive care unit at Hull Royal Infirmary said it is 'impossible to describe how hard the working environment is right now' as she revealed staff have had to care for patients who have died without their family by their side. Some have contracted coronavirus themselves.        With families shut out of intensive care, nurse Luke works on a ward where he has to choose whose hand to hold in their final moments, and who will have to die alone. | 'I knew one of the patients had some video calls from their family, so I contacted his family and gently told them he'd be passing away imminently.' 'I propped up an iPad so his daughter could speak to him on FaceTime until the end. She was saying, 'It's OK, Dad - you'll soon be in heaven with Mum'. My goggles filled with tears    Then on top of that I've used all the basic things - mouth care is so important and just making sure their positioning is right and actually, just holding their hands. Making sure there's a staff member to hold their hand, because I can imagine it's incredibly scary for people as they are gasping for air, they've got no relatives around, it's a really horrible situation. | 'On the bed next to him, I sat with an elderly gentleman who was passing away and we didn't have any family contacts for him. 'We never let anyone die alone. So I held his hand and told him how gorgeous the weather has been since lockdown, how the birds are singing louder, sunsets are more beautiful, skies are so clear and nice.”    Rebecca said, adding: 'One of my patients deteriorated really quickly and my role was to ensure I was providing comfort to the patient, who didn't have any relatives with them. |
| **Social Media:** | Negative sentiment: This was when the amount of daily deaths in both hospitals and care homes were at their peak, and health care workers and the media were sharing their worry about the lack of good quality end-of-life care for patients, who were unable to see their families before dying. This was labelled as "inhumane", with nurses and care home staff also reporting low morale on Covid-19 wards.    TWEET: “My sister 67, a nurse on the front line feels she is no more special & deserving than all the other key workers. She is on a non COVID ward looking after palliative patients of all ages & struggles with wearing PPE when patients are dying [broken heart emoji]” – 02/05/2020 | Positive sentiment: Shared around discussion of the actions of key organisations such as Hospice UK, who were working to help improve the experience of patients and bereaved families. There were also positive reports of FaceTime being used to link dying patients with families in their final moments.          TWEET: [picture of flowers] received these today from a relative to say thank you for looking after her Uncle at End of Life and providing daily updates via the telephone. #COVID19 has made EOL particularly difficult, but this is why I always try my best” – 22/06/2020 | Positive sentiment:  healthcare workers shared learning resources across international borders, in a series of conversation threads on Twitter, where UK healthcare workers shared guidance from Italian healthcare workers regarding having difficult Palliative/ end-of-life conversations with Covid-19 patients and their families. This included a useful infographic giving guidance about good and bad modes of interaction.    TWEET: #makethedifference |
| **Policy Review:**  Clinical guide for the management of palliative care in hospital during the coronavirus pandemic: Keeping the care in healthcare (version 2) | “Because of infection control measures the patient’s family members may not be able to attend or having to self-isolate or care for another sick individual” | “Support for those to the dying person, including the ability to keep in touch via phone or virtual communications (for example, Skype, WhatsApp)” | “As far as possible, try to make the immediate environment as conducive as possible to a peaceful and dignified death” |
| COVID-19: Personal Protective Equipment (PPE) Plan | “any clinician or care worker who is working in a hospital, primary care or community care setting, including care homes and delivery of home care who are within 2 metres of a possible or confirmed COVID-19 patient should wear an apron, gloves, surgical mask and eye protection, based on the risk of transmission.” |  |  |
| Clinical guide for supporting compassionate visiting arrangements for those receiving care at the end of life |  | “Sometimes a child dies very suddenly or unexpectedly. Healthcare teams always make every effort to ensure that a parent or carer can be present. It is important that families are reassured that staff will always comfort and care for a dying child in the very rare occasion when a parent or carer cannot be there.” |  |
| Interviews: | “At the same time, they're so important for the patients, to have someone supporting them, advocating for them and they were lacking that. So, that was pretty rough to kind of see them without that.” (Anaesthetist, Theatre)          “We were often very aware that we had these worried, frightened relatives who couldn’t visit and needed that proximity which we would normally encourage. They couldn’t come. (Nurse, Infectious Diseases)        “It does slightly influence what you do in terms of how you smile as much as anything else, cos you have to try and smile with eyes rather than just your mouth.” (Consultant, Paediatrics)  “never got to see all the important people around that person and how important that person was to other people” (Consultant, Palliative Care). | “Is actually quite good because we have an extra team which kind of deals with communication and an extra team of student doctors that is kind of in charge of the iPads. So they call the family, they set a time for the meeting the give the iPad and we put it just in front of the patient.” (Nurse, ICU)    “And there was other things as well, like there was some patients got like a knitted heart which was donated by somebody so they basically could actually have this heart with them as a sort of reminder of their family sort of thing. So there was some really kind of lovely acts of generosity and kindness from people sort of thinking about how they might be able to continue that connection and that link with their loved ones and things. So it’s been a sort of quite creative time.”(Nurse, Palliative Care) | “So he was really, really peaceful and didn't struggle for breath and died a few hours later really peacefully so I think he actually had, and I would say that for nearly all of them, they had good deaths because of what we did.” (Nurse, Palliative Care)          “And actually thought, now I've done some really good work today, and sort of going round, you know, and colleagues being, oh thank goodness, can I have your help with this patient, as well?” (Senior Registrar, Palliative Care)      “I’m their only point of contact with the human world….you need to make sure those conversations count, you’re like an extended family member to them cos you’re the only person they’ve seen all day. A lot of the elderly people don’t have mobile phones…. so you have to be that communication between them…..so they can see their relatives…” (Nurse, Infection Control) |
